# Current–voltage characteristics of single-molecule diarylethene junctions measured with adjustable gold electrodes in solution

**DOI:** 10.3762/bjnano.3.89

**Published:** 2012-11-26

**Authors:** Bernd M Briechle, Youngsang Kim, Philipp Ehrenreich, Artur Erbe, Dmytro Sysoiev, Thomas Huhn, Ulrich Groth, Elke Scheer

**Affiliations:** 1 Physics Department, University of Konstanz, D-78457 Konstanz, Germany; 2 Institute of Ion Beam Physics and Materials Research, Helmholtzzentrum Dresden-Rossendorf, D-01328 Dresden, Germany; 3 Chemistry Department, University of Konstanz, D-78457 Konstanz, Germany

**Keywords:** diarylethene, mechanically controllable break-junction, molecular electronics, photoswitching, single-molecule junctions

## Abstract

We report on an experimental analysis of the charge transport through sulfur-free photochromic molecular junctions. The conductance of individual molecules contacted with gold electrodes and the current–voltage characteristics of these junctions are measured in a mechanically controlled break-junction system at room temperature and in liquid environment. We compare the transport properties of a series of molecules, labeled TSC, MN, and 4Py, with the same switching core but varying side-arms and end-groups designed for providing the mechanical and electrical contact to the gold electrodes. We perform a detailed analysis of the transport properties of TSC in its open and closed states. We find rather broad distributions of conductance values in both states. The analysis, based on the assumption that the current is carried by a single dominating molecular orbital, reveals distinct differences between both states. We discuss the appearance of diode-like behavior for the particular species 4Py that features end-groups, which preferentially couple to the metal electrode by physisorption. We show that the energetic position of the molecular orbital varies as a function of the transmission. Finally, we show for the species MN that the use of two cyano end-groups on each side considerably enhances the coupling strength compared to the typical behavior of a single cyano group.

## Introduction

Charge transport in single-molecule devices is actively investigated with the aim to realize functional electronic circuits [1–4], such as switches [5], transistors [4,6] or storage devices [7]. Novel physical phenomena arise when the junctions are exposed to control schemes including electrochemical or electric-field gating [8–10], mechanical stretching [11–13], magnetic fields [14–17], and light irradiation [3,5,15,18]. Of particular interest are optically addressable molecules, the transport properties of which can repeatedly and reversibly be changed by irradiation with light pulses.

An example of these photochromic molecules is given by the class of diarylethenes. They consist of a core containing an aromatic ring that can be switched open or close by irradiation with photons of two distinct wavelengths. Upon this ring-opening/ring-closure reaction the conjugation of the electronic π-system and therefore the conductance is supposed to be strongly affected as well. This ring-opening/ring-closure reaction is accompanied by only a small geometrical change, which makes diarylethene molecules promising building blocks for optoelectronic applications [19–20].

Since electrical measurements of diarylethene molecules started, measurements of the charge-transport properties of molecular ensembles by using large-area samples [21], molecular networks with nanoparticle electrodes [18], atomic force microscope (AFM) [22], and carbon-nanotube electrode [23] techniques, as well as structural studies using scanning tunneling microscopy (STM) [24–25] have been performed successfully. In addition, mechanically controlled break-junctions (MCBJs) [5,12] and modified STM [26] techniques were applied to create single-molecular junctions. It has been argued that strong electronic coupling between electrodes and the switching core may block the switching procedure [5,27–29]. This strong coupling is supposed to be enhanced by the presence of sulfur atoms in the switching core.

For this study we chose the recently developed class of sulfur-free diarylethenes (with proper side-arms and end-groups) in which the thiophene rings have been replaced by furans that are assumed to be less prone to unspecific binding to the metal electrodes [30–31]. Recently, low-temperature measurements of the current–voltage characteristics of single-molecule diarylethene junctions have been reported [32]. By applying the resonant-level model, the level alignment and the coupling strength of the dominant current-carrying molecular orbital (frontier orbital) [12,33–34] has been determined. The authors found unexpected behavior in that, for conjugated diarylethenes, the level alignment in the open state is better, i.e., closer to the Fermi energy, as compared to the closed state. In order to test whether this unusual behavior is caused by particular conformations adopted at low temperatures and to exploit their stability at room temperature, we perform measurements on the same molecules but in a solvent environment. We introduce an additional member (MN) of the class that provides a narrow distribution of conductance values and therefore seems particularly suitable for applications.

## Results and Discussion

**Figure 1:**
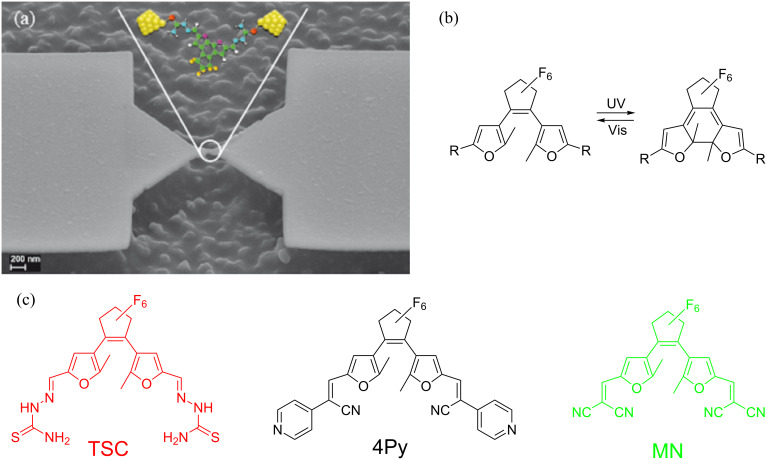
(a) Scanning electron micrograph of the MCBJ device with an illustration of a Au–4Py–Au junction. (b) Sketches of open (left) and closed (right) forms of photochromic molecules (difurylethene); R indicates the extended side-arms and end-groups. (c) Structures of three different molecules, 4Py (black), TSC (red), MN (green) investigated in this study.

We investigate charge transport through the species labeled TSC, 4Py and MN, shown in Figure 1c, contacted by adjustable Au electrodes (Figure 1a) in a MCBJ system operating at room temperature in a liquid environment [33,35–36]. The chemical synthesis together with their photochemical properties as analyzed by NMR and UV–vis measurements of TSC and 4Py, have been reported in [30]. For MN the respective data is given in Supporting Information File 1. The open and closed forms of diarylethene are shown in Figure 1b. The open isomer closes the central ring under UV light irradiation forming a completely π-conjugated molecule, while the closed isomer opens the ring under visible light irradiation, restricting the π-conjugation in the side-arms.

The optimum irradiation wavelengths for triggering the ring opening reaction vary slightly from species to species, because the wavelength of the absorption maximum is related to the extension of the π-system [30–32,37]. Out of the available members of this class of molecules we chose TSC because of the flexibility of its side-arms with the expectation that this species would adapt easily to varying electrode distances. Furthermore the two binding sites per end-group are expected to enhance the binding strength and therefore the robustness of the junctions.

4Py was selected because the low-temperature measurements revealed a narrow conductance distribution, a high switching ratio, and a relatively high conductance level in both states. Naively, the π-conjugation through the entire molecule in the closed form is supposed to show higher conductance than the broken π-system. While this has been confirmed by measurements in molecular ensembles and arrays [18,21] and single-molecule junctions [5,26], the underlying change of the electronic system appeared to be counterintuitive [32]. Namely, the current-carrying frontier orbital in the open state has shown to be better aligned with the Fermi energy (*E*_F_) than in the closed state. This well-aligned level, however, is coupled more weakly to the electrodes resulting in the lower conductance of the open isomer.

We use nano-fabricated MCBJ electrodes made of gold. An electron micrograph of a sample is shown on Figure 1a. Prior to mounting the nano-fabricated samples in a custom-designed MCBJ system with a pipette containing the molecular solution, the molecules are dissolved in toluene (Tol), isopropanol (IPA) or a mixture of 50% tetrahydrofuran and 50% toluene (THF/Tol) and switched to either the open or the closed form by irradiation at the suitable wavelength. TSC is investigated in both IPA and THF/Tol to test potential influence of the solvent on the transport properties [35–36]. Since we found no systematic differences, we here restrict ourselves to the data recorded in IPA.

Upon stretching of the metallic bridge, the last single-atom Au–Au contact breaks at a conductance *G* close to one conductance quantum ≈1 *G*_0_, with *G*_0_ = 2*e*^2^/*h*. Upon further stretching, one or several additional plateaus, corresponding to the conductance of a few or single-molecule contacts in different conformations, appear. Finally, the metal–molecule–metal contact breaks, and hence the conductance drops to below 10^−8^ *G*_0_. This is the lowest conductance value that we are able to detect, and it is one to two orders of magnitude smaller than the one that was achieved with the STM break-junction technique under similar conditions and at similar bias voltages [25]. This procedure is reversed, releasing the junction back until Au–Au contacts with a conductance of more than 100 *G*_0_ are achieved. For comparison we repeated these procedures for the solvents Tol and THF/Tol without molecules.

Typical conductance traces recorded at a DC voltage of 100 mV are displayed in the Supporting Information File 1. For the molecular solutions we calculate conductance histograms from ≈100 stretching and relaxing traces, and we find rather weak features in both forms (see Supporting Information File 1) as usual for room-temperature measurements in solution [26,35–36,38–39]. Under these conditions pronounced structures in the histograms are observed only when larger statistical ensembles of several thousands of traces are used. A more detailed discussion about the histograms and the stability of individual junctions is given in Supporting Information File 1.

For recording the current–voltage (*I*–*V*) characteristics the breaking procedure can be stopped at any position of the stretching or relaxing trace, meaning that the junctions are not necessarily in a stable position corresponding to a preferred conductance value. We ramp the voltage up to +1 V, then decrease it to −1 V and finally sweep it back to zero while the current is monitored. In most cases, the conductance at the end of the sweep is different from the initial one, indicating that the junctions relaxed into a more stable configuration during the sweep.

**Figure 2:**
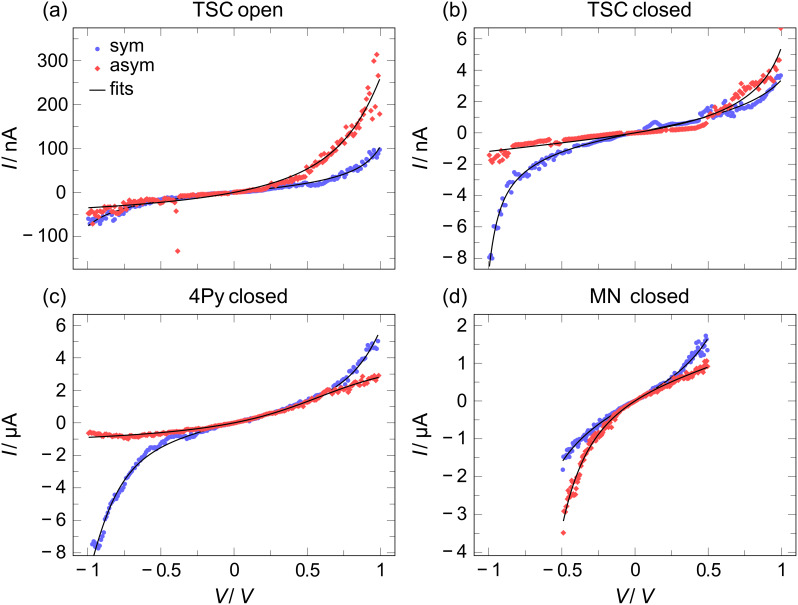
Examples of *I*–*V* characteristics of open and closed isomers. (a) Open form of TSC, (b) closed form of TSC. (c) Closed form of 4Py, (d) closed form of MN. The colored curves are experimental data, and the black curves are the fits to Equation 1.

We record up to several hundred *I*–*V*’s for each molecule in both states. However, only a limited subset of these shows the *s*-shape that is archetypical for molecular conduction. Other shapes include mainly jumps or kinks, presumably due to reorganizations of the contact geometry. The yield varies from species to species. It is highest for MN, intermediate for TSC, and lowest for 4Py (for details see Supporting Information File 1). Among the *s*-shaped curves we find symmetrical ones, i.e., the current amplitude at a given voltage is the same for both polarities of the bias, and asymmetric ones that resemble the *I*–*V*’s of a diode with higher current level in one polarity than in the other [38]. For the following analysis we restrict ourselves to those *I*–*V*’s that are stable during the complete voltage ramp, i.e., where no jumps or kinks occur. We evaluate both symmetric and asymmetric *I*–*V*’s. Examples of *I*–*V*’s taken on all three molecules are given in Figure 2.

For deducing a microscopic understanding of the charge transport through these molecules, we apply the single-level (resonant-level) transport model. The single-level model is applicable in the case of coherent transport and makes use of the Landauer picture [2,40], which describes the current as the energy integral over the transmission probability of a scatterer:

[2]
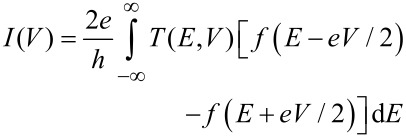


It assumes a single-molecular orbital at energy *E*_0_(*V*) coupled via the coupling constants Γ_L_ and Γ_R_ to the left and to the right leads. The coupling results in a broadening of the level and yields a resonance with Lorentzian shape for the transmission function *T*(*E*,*V*) [2,12,32–34,41].

[1]
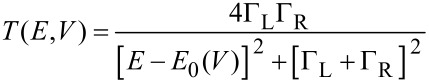


In the case of asymmetric coupling, i.e., Γ_R_ ≠ Γ_L_, the position of the energy level is a function of the applied voltage. We assume that the voltage drops at the left and right interface according to the coupling rates:

[3]
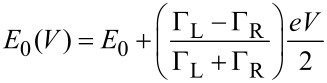


In general, the dominating molecular orbital that carries the current is formed by either the HOMO or the LUMO of the molecule coupled to the electrodes: *E*_0_ = |*E*_F_ − *E*_HOMO or LUMO_|; *E*_F_ is the Fermi level, and *E*_HOMO or LUMO_ is the energy level of the HOMO or LUMO. Each *I*–*V* curve was fitted with this model, and the energy level and the level broadening were inferred from these fits. The fitting procedure and criteria for successful fitting are described in the Supporting Information File 1. In the case of single-channel conduction the transmission corresponds to the linear conductance in units of the conductance quantum *G*_0_, *G*/*G*_0_ = *T*(*E*,0) = *T*.

A considerable part of the measured *I*–*V*’s can successfully be described with the single-level model, but only when considering different coupling constants Γ_R_ ≠ Γ_L_ in Equation 1. Strictly speaking for all *I*–*V*’s the fitting parameters are slightly asymmetric because of measurement noise limiting the precision of the fitting procedure. In what follows we refer to those *I*–*V*’s as symmetric when the asymmetry ratio α = Γ_R_ / Γ_L_ is in the range of 0.5 ≤ α ≤ 2, and as asymmetric when the ratio is outside this range.

**Figure 3:**
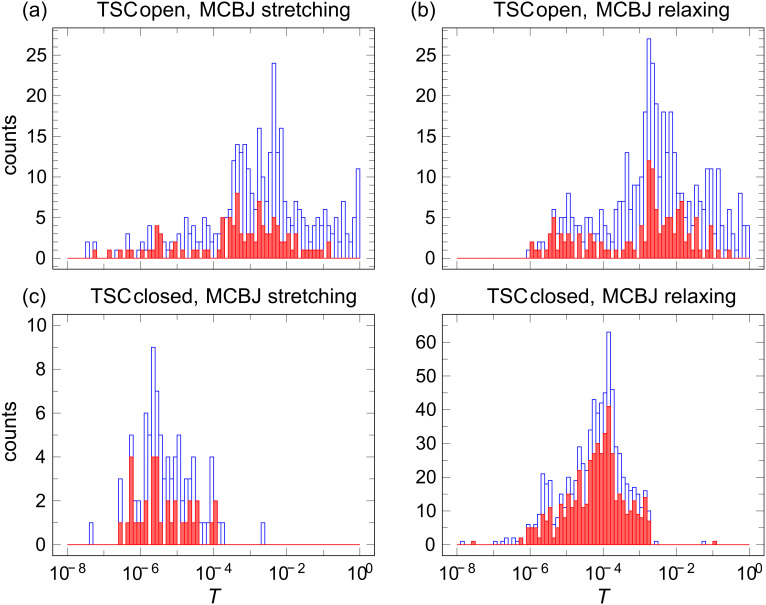
Transmission histograms of TSC. The open columns represent the transmission values from all *I*–*V*’s, the closed columns only from those *I*–*V*’s that are successfully described by the single-level model (symmetric as well as asymmetric). Panels (a) and (b) are for the closed isomer, panels (c) and (d) for the open isomer. The data shown in panels (a) and (c) were recorded during stretching of the junctions, the data in panels (b) and (d) during relaxing of the junctions.

A significant number of the *I*–*V* curves cannot be fitted by Equation 1 at all. They are discarded for further analysis. We attribute those *I*–*V*’s to multimolecule contacts, metallic tunnel contacts, or just instable contacts due to unspecific binding. This interpretation is supported by the further analysis of the conductance (transmission) histograms. As an example we plot in Figure 3 the conductance histograms calculated from the zero-bias conductance over all *I*–*V*’s recorded on the species TSC (blue columns). The red columns show the subset of these data that are successfully fitted with the single-level model. For completeness we separate data recorded when stretching or when relaxing the junction, although no pronounced differences are observed.

The first remarkable observation is that there are no *I*–*V*’s in the closed state at transmissions above 0.002 *G*_0_. When attempting to record *I*–*V*’s in this range the junctions jumped immediately to either a higher or a lower conductance. From the usual conductance histogram (i.e., recording of the linear conductance during continuous stretching or relaxing of junctions without interruptions for recording *I*–*V*’s, as shown in Supporting Information File 1), we observed a minimum in the histogram around 10^−2^ *G*_0_ and a strong increase of counts above this value. Our analysis of the *I*–*V*-based histograms clearly shows that these highly conductive counts do not reflect single-molecule contacts.

When investigating the open state, many *I*–*V*’s were found at high conductance with a clear maximum around 10^−3^ *G*_0_ and a weaker one around 10^−5^ *G*_0_. Again, not all of these were successfully described by the single-level model. In particular, *I*–*V*’s with *G* > 10^−2^ *G*_0_ could barely be fitted successfully. Apparently, these junctions are not single-molecule junctions either. The fact that *I*–*V*’s with higher transmission can be found for the open form can be explained with the weaker steric hindrance that enables the formation of a stable contact even though the gap of the electrodes may not have the ideal spacing.

These findings are further supported by the observation of asymmetric *I*–*V* curves with rather high likelihood (see below) while at low temperatures the stable contacts are preferentially symmetric [32]. Unequal coupling constants reflect asymmetric voltage drops at the interfaces of the molecule to the metal electrodes. These may arise from either different binding positions of the molecular end-group to the metal electrode or different degrees of conjugation of the side-arms. Both options are more likely to occur at higher temperature than at low temperature because of the high mobility of the metal-electrode atoms and thermal energy.

**Figure 4:**
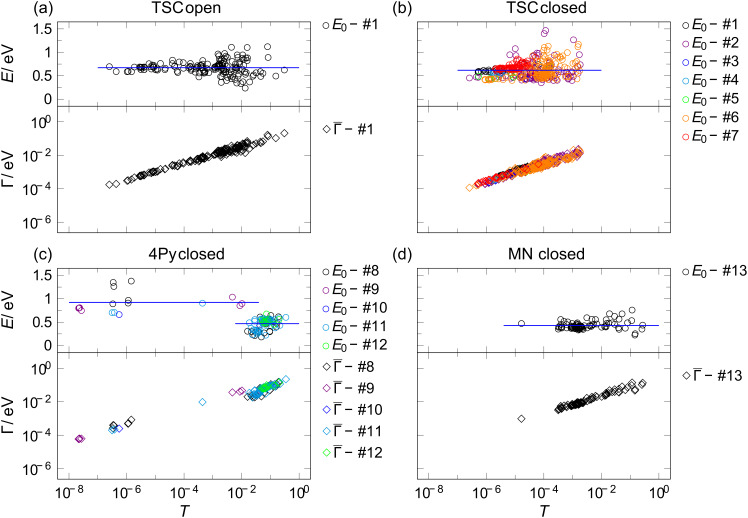
Fitting parameters Γ and |*E*_0_| for (a) open form of TSC, (b) closed form of TSC, (c) closed form of 4Py, and (d) closed form of MN as a function of the transmission for symmetric *I*–*V*’s. The various colors in panels (b) and (c) refer to the individual samples used for recording the data. We define those *I*–*V*’s as “symmetric” for which the ratio of Γ_L_ to Γ_R_ is between 0.5 and 2.

**Figure 5:**
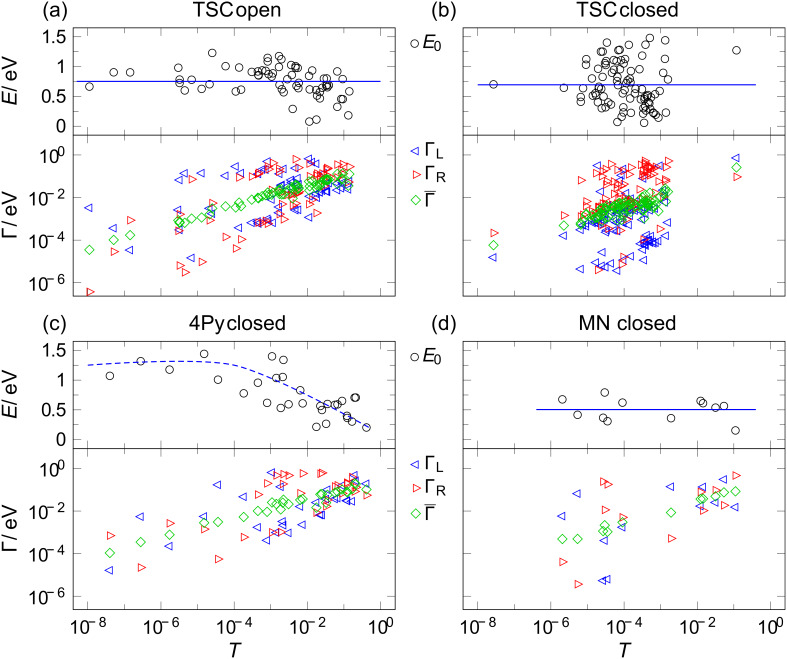
Fitting parameters Γ_L_ and Γ_R_ and |*E*_0_| for (a) open form of TSC, (b) closed form of TSC, (c) closed form of 4Py, and (d) closed form of MN as a function of transmission for asymmetric *I*–*V*'s.

The values extracted from fitting the single-level model for all molecules are plotted in Figure 4 for the symmetric case and in Figure 5 for the asymmetric case as a function of the transmission. We first discuss the symmetric *I*–*V*’s of the species TSC. As mentioned before, higher transmissions are achievable (up to 0.1) in the open state than in the closed state, where we find *T* < 10^−3^. At first sight this behavior differs from the low-temperature results where a preferred conductance value of 8 × 10^−7^ *G*_0_ was found for the closed form and 7 × 10^−8^ *G*_0_ for the open form for Au–TSC–Au single-molecule junctions at low temperature and in vacuum. However, this difference can be attributed to the different binding situations under the experimental conditions probed here.

In the present experiment, for recording the *I*–*V*’s we do not concentrate on the preselected preferred conductance values deduced from histograms, but rather probe the whole transmission range for stable junctions. Once a stable junction is formed at low temperature, its lifetime amounts to hours, while at room temperature in solution it is limited to a couple of minutes. By the present method we are thus addressing a broader ensemble of junctions than are accessible at low temperature. As a result we observe a rather continuous distribution of transmission values, presumably because the higher mobility of the atoms in the metal electrodes at room temperature and in solution enables the formation of a contact more easily.

As can be seen from Figure 4a, Figure 4b, Figure 5a and Figure 5b, a wide range of transmissions is achieved in both isomers with a systematic increase of the coupling constants Γ with the transmission and no systematic dependence of *E*_0_ on the transmission. The averaged values of *E*_0_ and Γ for both isomers of all species are summarized in Table 1. In Figure 4 we show the results for the closed forms of TSC and 4Py color-encoded with respect to the individual electrode samples. No sample-dependence is observed.

**Table 1:** Molecular energy level |*E*_0_| as plotted in Figure 4 and Figure 5 averaged over the indicated transmission ranges. Because of the systematic variation of |*E*_0_| with *T* for the species 4Py, we calculated separate average values for the low *T* < 10^−2^ and the high *T* > 10^−2^ range. The *I*–*V*’s are categorized as symmetric when the ratio α = Γ_R_/Γ_L_ is in the range of 0.5 ≤ α ≤ 2, and as asymmetric when the ratio is outside this range. The errors are the standard deviations of the averaging procedure.

species	transmission range	isomer	symmetry	number of fittable *I*–*V*’s	*|E*_0_*|* (eV)

TSC	10^−8^ < *T* < 10^−1^	open	sym	164	0.67 ± 0.14
TSC	10^−8^ < *T* < 10^−1^	open	asym	65	0.75 ± 0.24
TSC	10^−8^ < *T* < 10^−3^	closed	sym	377	0.62 ± 0.16
TSC	10^−8^ < *T* < 10^−3^	closed	asym	88	0.69 ± 0.37
MN	10^−6^ < *T* < 10^−1^	closed	sym	86	0.43 ± 0.09
MN	10^−6^ < *T* < 10^−1^	closed	asym	12	0.51 ± 0.18
4Py	*T* < 10^−2^	closed	sym	13	0.92 ± 0.21
4Py	*T* < 10^−2^	closed	asym	7	0.98 ± 0.30
4Py	*T* > 10^−2^	closed	sym	100	0.47 ± 0.11
4Py	*T* > 10^−2^	closed	asym	14	0.48 ± 0.18

Since the analysis of the *I*–*V*’s does not provide information about the sign of *E*_0_ we cannot reveal whether the current-carrying orbital is the LUMO or the HOMO. We therefore restrict our analysis to the absolute value of *E*_0_. Since we do not expect the dominant orbital to fluctuate arbitrarily between HOMO and LUMO transport upon slight changes of the coupling constant, we interpret our data such that the current-carrying molecular orbital is the same throughout the whole range of transmissions. The closed isomer features a wider distribution of *E*_0_ values than the open one. The average *E*_0_ in the closed state amounts to 0.62 eV for the symmetric *I*–*V*’s and 0.69 eV for asymmetric ones. It is slightly smaller than in the open state, where we find *E*_0_ = 0.67 eV for symmetric and 0.75 eV for asymmetric *I*–*V*’s, in agreement with the simple expectation that the closed state should have a smaller HOMO–LUMO gap and if no charging occurs (i.e., the level alignment remains the same), one should expect the frontier orbital to be located closer to *E*_F_. However, this observation is in partial disagreement with the findings of Kim et al. who found the values 0.6 eV (closed form) and 0.41 eV (open form) at low temperatures [32].

The rather similar values found for the closed state at low and at room temperature suggest that although the stability conditions at room temperature seem to differ from those at low temperature, the current-carrying molecular orbital may be the same. The pronounced difference in the open state implies that most likely in the open form another conformation is adopted under the environmental conditions of the present study. The rather wide-spread distribution of *E*_0_ in the closed state is in agreement with the findings at low temperature, in which two preferred closed-state configurations were reported [32]. For symmetric coupling the *E*_0_ values are smaller than in the open state, in accordance with the simple expectation that symmetric coupling should improve the level alignment.

In order to probe the influence of the solvent we repeated the experiment with the molecules being dissolved in a mixture of THF and toluene. Within our limited statistics we found no systematic difference between both data sets.

The analysis of the asymmetric curves reveals a very pronounced asymmetry with coupling ratios of up to 10^3^ in the low-transmission regime. A possible interpretation of these very asymmetric curves is that the molecule is chemisorbed to one electrode only and physisorbed to the other one. While there is no strict distinction between physisorption and chemisorption, we use these terms for describing strong coupling including a bond formation (chemisorption), and van der Waals like coupling (physisorption).

In the case of physisorption, one can expect that the current is mediated by tunneling. For thiol end-groups on gold, it has been shown that both chemisorption and physisorption is possible [42–44] depending on the surface morphology and the deposition method. For the amine end-group the experimental situation is not so clear. However, since in most studies in which molecules with the same molecular core but different end-groups are compared, a higher conductance is found for thiol-terminated molecules than for amine-terminated [39,45], we expect that in the asymmetric junctions the coupling on one side is realized through the thiol end-group and on the other side-arm through the amine end-group.

We now discuss our findings and data analysis of the species 4Py, which we probed in the closed state only. Examples of *I*–*V*’s and their fittings for the symmetric and the asymmetric case are shown in Figure 4c and Figure 5c. For 4Py we find a very broad range of transmissions ranging from 10^−8^ to 10^−2^. We find symmetric as well as asymmetric *I*–*V*’s, but only very few symmetric ones in the intermediate range of ≈10^−4^, pointing to two qualitatively different junction geometries, one with weak coupling and one with strong coupling. The weakly coupled symmetric curves feature a typical coupling strength of 10^−4^ eV, while the strongly coupled have Γ ≈ 10^−2^ eV. *E*_0_ is also different in these two ranges: it is around 1 eV in the weakly transmitting junctions and around 0.47 eV for the better conducting junctions but with a rather broad distribution.

Since these highly conductive junctions show preferentially symmetric *I*–*V*’s, a symmetric coupling situation on both electrodes can be expected. We thus interpret the weakly conducting junctions as being physisorbed at the metal electrodes while the highly conductive ones are chemically bound. This is in agreement with the findings that nitrogen ended molecules may have a lower likelihood to bind chemically, but if chemical bonds are formed, the coupling strength may be higher than the one of the thiol end-group, because the pyridine ring is part of the π-conjugated side-arms [32,38]. Indications for multiple binding sites and switching between these sites have been reported before [46–47]. In particular, when comparing our findings with the low-temperature measurements on the same molecule, we find a similar value of *E*_0_ ≈ 0.47 eV suggesting that the same molecular orbital carries the current as observed for the closed state of TSC [32].

A striking observation is made for the asymmetric curves: here we find a continuous variation of transmission and a systematic decrease of *E*_0_ in the same range from ≈1 eV to ≈0.3 eV with increasing Γ and *T*. A possible interpretation of this observation is again that one end is chemisorbed, while the other one is physisorbed forming a tunnel contact to the gold electrode [35]. When enhancing the coupling strength of the weak bond continuously, the molecular orbital shifts continuously as well.

Finally, we briefly discuss the findings on MN, where we also restricted ourselves to the closed state. Here we find rather high transmissions ranging from 10^−4^ to 10^−1^ with a much higher probability of symmetric curves than asymmetric curves, presumably because of the high symmetry of the side-arms with two equal end-groups on each arm. This structural feature seems to be favorable for adapting to the variable surface geometry of the electrodes. Accordingly *E*_0_ is rather small *E*_0_ ≈ 0.43 eV indicating good level alignment, and it remains constant over the whole range while Γ_L_ and Γ_R_ increase with increasing *T*. The cyano end-group seems to favor chemisorptions when assembling monolayers on well-defined flat surfaces [48–49]. In single-molecule contacts, however, the cyano group has been shown to give rise to rather low-conductance junctions [33,38,41,50]. The double CN-motif used here seems to provide stronger coupling and considerable improvement of the level alignment. Since no low-temperature measurements have been performed on this compound, we cannot compare the absolute values in a more detailed manner.

## Conclusion

We have investigated charge-transport characteristics of photochromic molecules using the MCBJ technique in a liquid environment at room temperature. We have investigated three different diarylethene molecules with a sulfur-free switching core to reduce the possibility of unspecific binding. The conductance has been examined during repeated breaking and forming of the atomic contacts. By analyzing the *I*–*V* curves within the framework of the single-level transport model, we are able to identify those contacts that are indeed formed by a single molecule. Under these conditions also asymmetric coupling situations can be achieved that can be explained by physisorption of one of the end-groups to the electrodes. Rather high transmissions in the range of 10^−4^ to 10^−3^ can be adjusted. We demonstrate that for molecules that are known to preferentially chemisorb on gold, the change of transmission is mainly achieved by tuning the coupling of the molecular orbital to the metal electrode while the dominant transport level *E*_0_ remains mainly constant. For molecules that favor physisorption, both the coupling and the energy of the frontier orbital can be tuned. These findings are important for the further improvement of photochromic molecules in future molecular electronic devices.

## Experimental

### Synthesis of molecules

The starting materials were purchased from Acros, Sigma-Aldrich, Fluka, Fluorochem, ABCR and Alfa Aesar. Compound **1** was synthesized as described in the literature [30]. Column chromatography was performed on MN Kieselgel 60 M (silica gel, 40–63 μm 230–400 mesh ASTM, Macherey-Nagel, Düren, Germany). TLC was performed on Polygram Sil G/UV_254_ (0.2 mm of silica gel, Macherey-Nagel, Düren, Germany). An UV lamp (254 nm) was used for detection. Elemental analyses were performed on a CHN-analyzer Heraeus (CHN-O-RAPID) by the Microanalysis laboratory of Konstanz University. Analytical HPLC was performed on Merck RP-18 column (250 × 4.1 mm) by using gradient or isocratic eluation with acetonitrile–water mixture (UV detection at 254 nm). GC/MS was performed on an Agilent GC/MS 7890A/5975C instrument (EI, 70 eV). HRMS ESI/FT-ICR spectra were recorded on a Bruker APEX II FT/ICR instrument. FABMS was performed on a Finnigan MAT 8200 instrument. MALDI–TOF spectra were recorded on a Bruker Biflex III instrument with a pulsed nitrogen laser (337 nm). IR spectra were recorded on a Perkin-Elmer 100 Series FT–IR spectrometer. UV–vis spectra were recorded on a Cary 50 spectrophotometer (Figure S1 in Supporting Information File 1). NMR spectra were recorded on a Bruker Avance DRX600 (600 MHz) and a Jeol ECP-Eclipse 400 (400 MHz).

### Device fabrication

The spin-coating of polyimide (2 μm in thickness) was performed on a softly polished bronze wafer (200 μm in thickness), and then the wafer was annealed for 6 h at 430 °C in vacuum (10^−5^ mbar). The polyimide layer serves as an electrical insulator and a sacrificial layer in the subsequent etching process. Prior to performing the electron beam lithography process, a double layer of electron-beam resists, MMA-MAA/PMMA, was deposited by spin-coating on the wafer. After developing, the patterned samples were mounted in an electron-beam evaporator under ultrahigh vacuum (10^−9^ mbar), and gold of about 80 nm thickness was deposited at a rate of 1 Å/s. After lift-off, the polyimide layer was partially etched away (thickness reduction ≈700 nm) by employing O_2_ plasma in the vacuum chamber of a reactive ion etcher, in order to form a free-standing bridge [51–52].

### Break-junction setup for measurements of molecular contacts in solution

**Figure 6:**
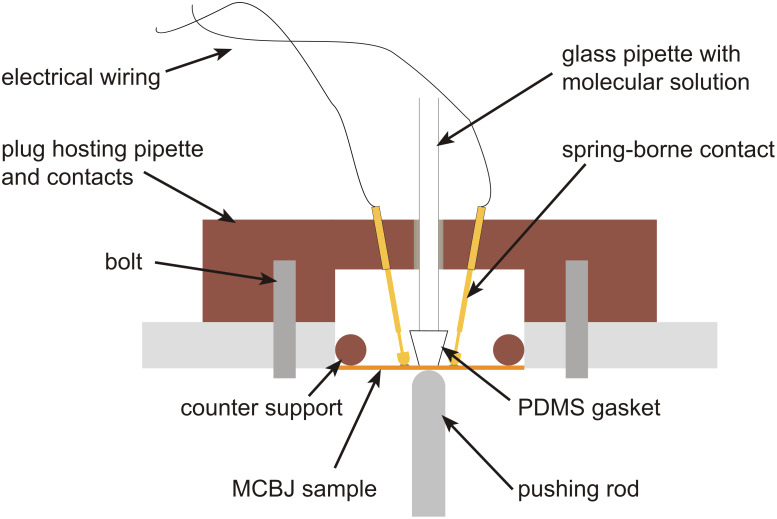
Break-junction setup for use in liquid environment. A PDMS-sealed glass pipette, in which the molecular solution circulates, is pressed onto the central part of the MCBJ chip with the help of a plug screwed to the sample holder. The electrical contacts are realized in this case through spring-borne contacts outside the gasket.

The samples were mounted onto the three-point bending mechanism shown in Figure 6. The electrodes were contacted by lowering the spring-borne contacts onto the pads. Before assembling the molecules, the open or the closed form of the switching core was initialized by irradiating with visible or UV light, respectively. A dilute solution of molecules (≈10^−4^ M) in 5 mL of the respective solvent was prepared and transferred into the PDMS sealed pipette and carefully lowered onto the electrode device [36]. The setup was installed in a closed metal case for shielding high-frequency noise and for avoiding undesired illumination of the molecules.

### Electrical measurement

All electrical measurements were performed at room temperature in a liquid environment. The conductance measurements of stretching and relaxing were performed by a sub-femtoamp source-meter (Keithley 6430) operating with an automatic variable-gain preamplifier. The same instrument was used to measure the *I*–*V* curves. The voltage was swept at a rate of 100 mV/s. Every ground of the system was carefully designed to avoid ground loops and electrical noise. All data were collected by a Labview program through GPIB cables.

## Supporting Information

File 1 Additional experimental details, data, fitting parameters and figures.
Supporting information gives detailed information about the synthesis of molecules, sample calibration and statistics, stretching and relaxing curves, histograms of stretching and relaxing curves, current–voltage characteristics of TSC in THF/Tol, and best-fit parameters of all current–voltage characteristics displayed.
