# Supplementary material for: Current–voltage characteristics of single-molecule diarylethene junctions measured with adjustable gold electrodes in solution
Source: Beilstein J Nanotechnol. 2012 Nov 26;3:798–808. doi: 10.3762/bjnano.3.89 (PMC3554105; doi:10.3762/bjnano.3.89)
Supplement: File 1 — Additional experimental details, data, fitting parameters and figures. Supporting information gives detailed information about the synthesis of molecules, sample calibration and statistics, stretching and relaxing curves, histograms of stretching and relaxing curves, current–voltage characteristics of TSC in THF/Tol, and best-fit parameters of all current–voltage characteristics displayed. [file Beilstein_J_Nanotechnol-03-798-s001.pdf]

Supporting Information

for

Current–voltage characteristics of single-molecule diarylethene junctions measured with adjustable gold electrodes in solution

Bernd Michael Briechle[1], Youngsang Kim[1,§], Philipp Ehrenreich[1], Artur Erbe[2], Dmytro Sysoiev[3], Thomas Huhn[3], Ulrich Groth[3] and Elke Scheer*[1]

Address: [1]Physics Department, University of Konstanz, D-78457 Konstanz, Germany, [2]Institute of Ion Beam Physics and Materials Research, Helmholtzzentrum Dresden-Rossendorf, D-01328 Dresden, Germany and [3]Chemistry Department, University of Konstanz, D-78457 Konstanz, Germany

[§]now at: Department of Mechanical Engineering, University of Michigan, Ann Arbor, Michigan 48109, USA

Email: Elke Scheer* - elke.scheer@uni-konstanz.de

* Corresponding author

Experimental details, control experiments, and fitting parameters

Contents

page number

1. Synthesis data of molecules S2
2. Sample calibration and statistics S6
3. Stretching and relaxing curves S9
4. Histograms of stretching and relaxing curves S10
5. Testing the fitting procedure S12
6. Current–voltage characteristics of the solvents Tol and THF/Tol S13
7. Current–voltage characteristics of TSC in THF/Tol S15
8. Best-fit parameters of all current–voltage characteristics displayed in the manuscript and the Supporting Information S16
9. X-ray single crystal structure determination S17
10. References S24

1. Synthesis data of molecules

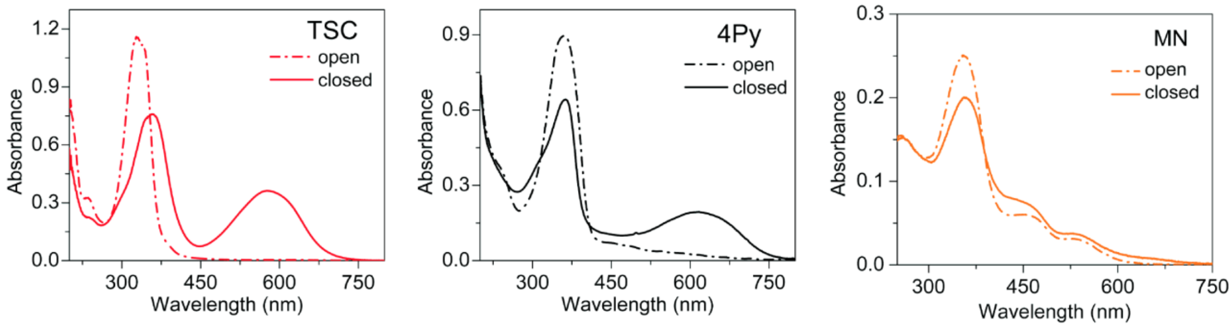

Figure S1: UV–vis absorption spectroscopy is performed for the “open” and the photostationary state labeled “closed” form of TSC, 4Py, and MN molecules. For TSC it could be shown by analysis of the NMR spectra that the photostationary state consists of molecules in the closed form only. For 4Py the photostationary state shows a clear long-wavelength absorption maximum at ~630 nm. For the closed form of MN we do not see a clear maximum at long wavelengths. Nevertheless, in solution it can be switched reversibly between the open state and a well-defined photostationary state. For initializing the closed state we irradiated the molecules with either 366 nm (MN,4Py) or 313 nm (TSC).

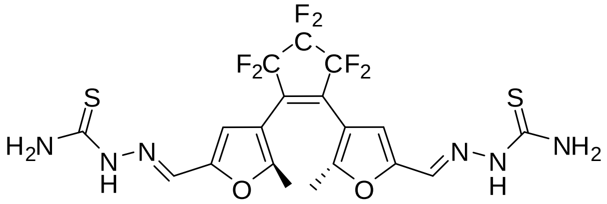

1,2-Bis{2-methyl-5-[(*E*)-(2-thiocarbamoylhydrazono)methyl]furan-3-yl}perfluoro-cyclopentene (**2**), (**TSC**): **1** (50 mg, 0.13 mmol) and thiosemicarbazide (23 mg, 0.26 mmol) were dissolved in hot EtOH (10 mL) with a few drops of pure acetic acid. The reaction mixture was stirred for 24 h at rt and was poured into water (30 mL) and filtered. After washing with cold EtOH (3 mL) and drying, 61 mg of dark-brown crystals were obtained. Yield: 89%; mp 203 °C; ^{1}H NMR (open form) (600 MHz, DMSO-d_6, 25 °C): δ 1.95 (s, 6H; CH_3), 7.07 (s, 2H; Ar-H (furyl)), 7.74 (br, 2H), 7.89 (s, 2H; *H*C-6), 8.25 (br, 2H), 11.50 ppm (br, 2H; *H*NN); ^{1}H NMR (open form) (400 MHz, methanol-d_4, 25 °C): δ 2.05 (s, 6H; CH_3), 6.92 (s, 2H; Ar-H (furyl)), 7.81 ppm (s, 2H; *H*C-6); ^{1}H NMR (closed form) (400 MHz, methanol-d_4, 25 °C): δ 1.64 (s, 6H; CH_3), 6.44 (s, 2H; Ar-H

(furyl)), 7.70 ppm (s, 2H; *H*C-6); ^{13}C NMR (150 MHz, DMSO-d_6, 25 °C): δ 13.58 (CH_3), 110.68 (C-3), 110.84 (m, $CF_2\mathit{C}F_2CF_2$), 112.11 (C-4), 116.06 (tt, ^1J(C,F) = 254, ^2J(C,F)=24 Hz; $\mathit{C}F_2CF_2\mathit{C}F_2$), 131.80 (C-6), 132.16 (t, ^2J(C,F)=24 Hz; $\mathit{C}CF_2$), 149.62 (C-5), 155.14 (C-2), 177.83 ppm (C=S); ^{19}F NMR (376 MHz, DMSO, 25 °C): δ −109.37 (br, 4F; $C\mathit{F}_2CF_2C\mathit{F}_2$), −130.78 ppm (br, 2F; $CF_2C\mathit{F}_2CF_2$); IR ν: 3422, 3526, 3144, 2962, 1590, 1504, 1275 cm^{-1}; FABMS (*m*/*z*) 539 $[M + H]^+$, 462, 389; HRMS $[M + H]^+$ calcd for $C_{19}H_{16}F_6N_6O_2S_2$ 539.0753; found: 539.0750; elemental analysis calcd (%) for $C_{19}H_{20}F_6N_6O_4S_2$: [M + $2H_2O$]: C, 39.72; H, 3.51; S, 11.16; found: C, 39.58; H, 3.36; S, 11.23.

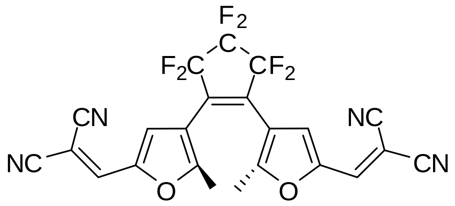

1,2-Bis[2-methyl-5-(2,2-dicyanovinyl)furan-3-yl]perfluorocyclopentene, (MN): To a solution of **1** (50 mg, 0.13 mmol) and of malodinitrile (18 mg, 0.28 mmol) in benzene (5 mL) was added piperidine (2 drops), and the mixture was stirred for 48 h. The organic solvent was removed in vacuo and the oily residue was filtered through a silica column with hexanes/EtOAc 1:1 as eluent. 13 mg of violet crystals were obtained. Yield: 21%; mp > 215 °C (decomp.); ^{1}H NMR (400 MHz, $CDCl_3$, 25 °C) δ 2.25 (s, 6H; CH_3), 7.25 (s, 2H; Ar-H (furyl)), 7.41 ppm (s, 2H, C-6); ^{13}C NMR (100 MHz, $CDCl_3$, 25 °C) δ 14.40 (CH_3), 79.94 (C-7), 110.60 (m, $CF_2\mathit{C}F_2CF_2$), 112.32 (CN (*trans* to *H*C-6), 113.20 (C-3), 113.35 (CN (*cis* to *H*C-6), 115.66 (tt, ^1J(C,F)=256, ^2J(C,F) = 24 Hz; $\mathit{C}F_2CF_2\mathit{C}F_2$), 122.27 (C-4), 133.14 (t, ^2J(C,F) = 24 Hz; $\mathit{C}CF_2$), 141.89 (C-6), 147.69 (C-5), 160.43 ppm (C-2); ^{19}F NMR (376 MHz, $CDCl_3$, 25 °C) δ −109.99 (t, ^3J(F,F) = 4.8 Hz, 4F; $C\mathit{F}_2CF_2C\mathit{F}_2$), −131.47 ppm (m, 2F; $CF_2C\mathit{F}_2CF_2$); correlations confirmed by ^{1}H-^{13}C-HSQC and ^{1}H-^{13}C-

HMBC (*cis*-^3J(H,CN) = 7.2 Hz, *trans*-^3J(H,CN) = 13 Hz) experiments; IR ν: 3059, 2237 (C≡N), 2225 (C≡N), 1615 cm^{-1}; FABMS (*m*/*z*): 511 $[M + Na]^+$, 489 $[M + H]^+$.

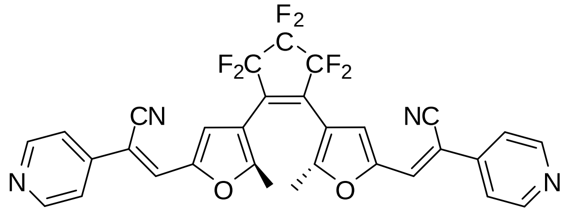

1,2-Bis(2-methyl-5-{(*Z*)-[2-cyano-2-(pyridin-4-yl)vinyl]}furan-3-yl)perfluorocyclopentene , (4Py): **1** (0.1 g, 0.25 mmol) and pyridine-4-acetonitrile hydrochloride (0.08 g, 0.5 mmol) were dissolved in dry MeOH (10 mL). After addition of K_2CO_3 (0.176 g, 1.27 mmol) the reaction mixture was stirred for 48 h at rt. After addition of diethylether (50 mL) the mixture obtained was washed consecutively with water (30 mL), aqueous K_2CO_3 (30 mL) and again with water (30 mL). Organic fraction was dried with $MgSO_4$, the solvent removed in vacuo and the dark residue purified by filtering through a silica column with hexanes/EtOAc 1:1 as eluent, thus giving 0.12 g of brown crystals. Yield: 77%. mp 182 °C; ^{1}H NMR (600 MHz, $CDCl_3$, 25 °C) δ 2.24 (s, 6H; CH_3), 7.24 (s, 2H; Ar-H (furyl)), 7.47 (s, 2H; C-6), 7.51 (dd, ^3J(H,H) = 4.6, ^4J(H,H) = 1.7 Hz), 4H; C-9), 8.69 ppm (dd, ^3J(H,H) = 4.6, ^4J(H,H) = 1.7 Hz), 4H; C-10); ^{13}C NMR (150 MHz, $CDCl_3$, 25 °C) δ 14.02 (CH_3), 106.63 (C-7), 110.82 (m, $CF_2\mathit{C}F_2CF_2$), 112.30 (C-3), 115.90 (m, $\mathit{C}F_2CF_2\mathit{C}F_2$), 116.31 (CN), 117.23 (C-4), 119.60 (C-9), 128.91 (C-6), 132.90 (t, ^2J(C,F) = 24 Hz; $\mathit{C}CF_2$), 140.75 (C-8), 149.07 (C-5), 150.78 (C-10), 157.01 ppm (C-2); ^{19}F NMR (376 MHz, $CDCl_3$, 25 °C) δ −109.94 (m, 4F; $C\mathit{F}_2CF_2C\mathit{F}_2$), −131.46 ppm (m, 2F; $CF_2C\mathit{F}_2CF_2$); IR ν: 3048, 2216 (C≡N), 1591, 1415, 1275 cm^{-1}; FABMS (*m*/*z*): 592 $[M^+]$, 506; HRMS calcd for $C_{31}H_{18}F_6N_4O_2$ $[M + H]^+$: 593.1407; found: 593.1394.

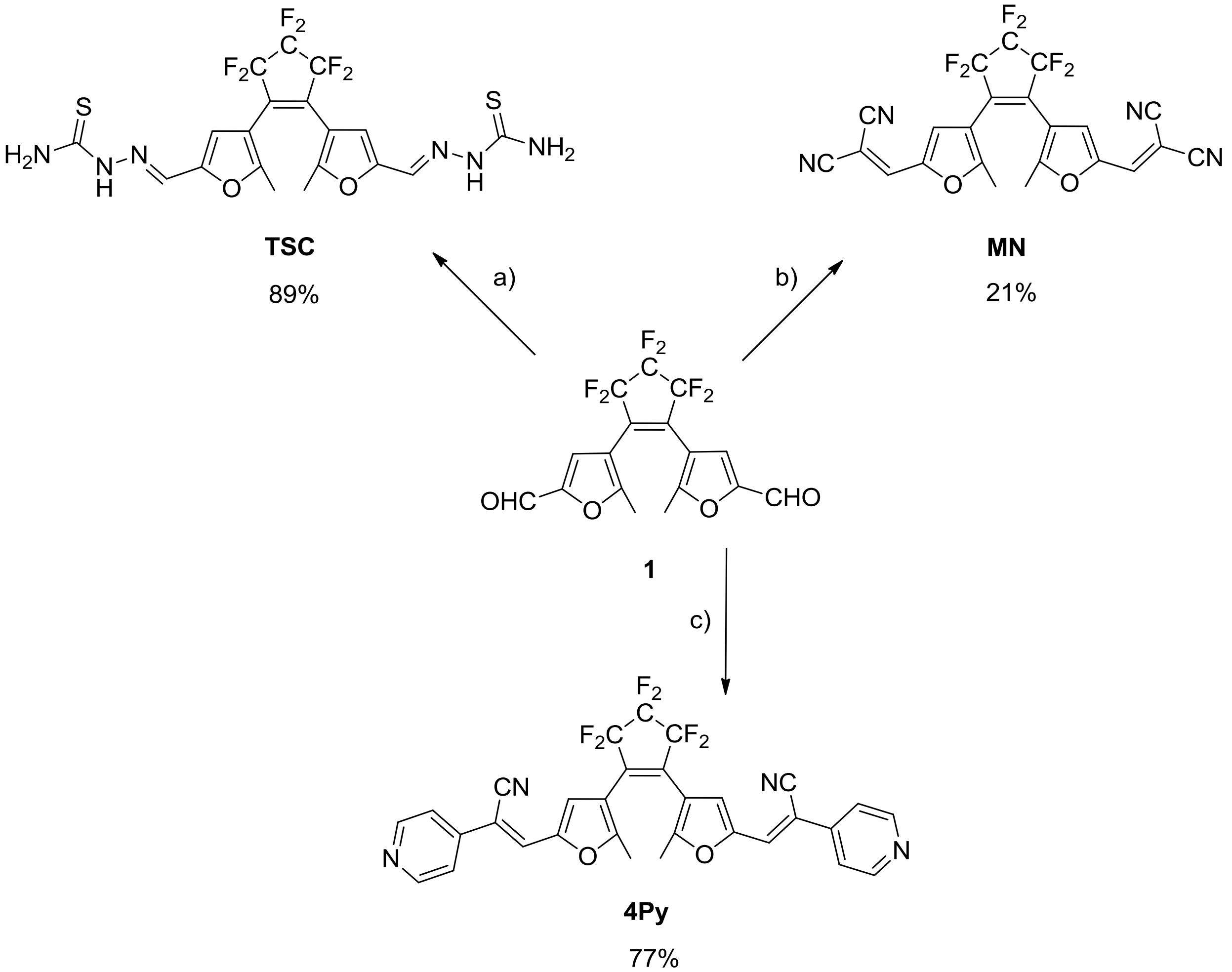

Scheme S1: Synthesis of functionalized molecular switches TSC, MN, and 4Py: (a) thiosemicarbazide, MeOH, rt, 24 h; (b) malodinitrile, piperidine, benzene, rt, 48 h; (c) 2-(pyridine-4-yl) acetonitrile hydrochloride, K_2CO_3, MeOH, rt, 48 h.

2. Sample calibration and statistics

The data presented in the manuscript have been recorded from a total of approximately 50 break-junction samples. Out of these samples roughly 20 were used for distance calibration in air and the various solvents. We recorded stretching and relaxing curves and determined the distance scale by fitting to the functional dependence for vacuum tunnelling along the lines of Grüter et al. [S1]. Our break-junction setup is designed to achieve a maximum life-time of single-molecule junctions. A drawback of this high-stability design is that it enables only rather slow stretching and relaxing speeds on the

order of 0.1 nanometres per second, at most. This enhances measuring time to a few minutes per stretching and relaxing cycle. Additionally, the rather soft polyimide insulation layer between the metal substrate and metal electrodes undergoes a creeping motion that limits the total measuring time to a few hours. Because of the creeping, the bending angle at which a particular electrode distance is obtained drifts to higher values, such that the elastic deformation limit of the substrate is reached and no further bending is possible. These two factors restrict the total number of deformation cycles per sample to a few hundred.

For measuring the molecular contacts we used fresh samples with nominally the same geometry assuming the same mechanical behaviour as in vacuum. This sets the error bar for the distance scale to roughly 30%. Finally, 22 samples were used to investigate the molecular contacts with varying lifetimes and therefore varying distribution over the individual species, (Table S1). We started the measurements by recording stretching and relaxing curves for training the electrodes and forming narrow metal tips, indicated by steps on the order of the conductance quantum when the conductance is on the order of a few quanta. This training is performed at air or directly with the molecular solution, because we observed an even stronger drift of the breaking point of the electrodes in the solvent. Simultaneously the shape of the current–voltage characteristics recorded in the solvent developed pronounced nonlinearities (see below). Both observations indicate the formation of disordered gold contacts and chains stabilized by incorporating carbon atoms resulting from the solvent [S2]. After this training a few hundred of these stretching/relaxing cycles were performed before we started recording the current–voltage characteristics (*I–V*'s). The described ageing effect limits the maximum amount of data that can be obtained from one sample. Because of this effect, it was not possible to establish single-molecule contacts that showed the typical s-shaped *I–V's* for all samples, hence reducing the number of

successful samples as indicated in Table S2. We stopped measurements of a given sample when no plateaus at 1 G_0 could be observed any more.

Table S1: Number of electrode devices used for the data reported in the manuscript. We usually started our measurements by recording stretching/relaxing curves and then continued with the measurement of the *I–V* measurements. For some devices only stretching/relaxing curves were recorded.

molecule	solvent	molecule state	# of samples with *I–V* measurements	total # of samples measured
TSC	IPA	open	1	2
TSC	IPA	closed	7	8
TSC	THF/Tol	open	4	7
TSC	THF/Tol	closed	3	13
MN	Tol	closed	1	5
4Py	Tol	closed	5	6

Table S2: Efficiency of the formation of molecular junctions for the three molecules under investigation measured by the relative number of *I–V* characteristics that can be described by the single-level model (“fittable”) with respect to the total number of *I–V*s recorded (“all”). The table shows accumulated data over all samples. The column headings “stretching” or “relaxing” indicate whether the *I–V*s have been recorded during bending or relaxing of the substrate.

molecule	solvent	state	stretching		relaxing	
			all	fittable	all	fittable
4Py	tol	closed	1206	110	156	35
TSC	IPA	closed	95	35	759	480
TSC	IPA	open	365	104	433	131
MN	tol	closed	86	60	61	41
TSC	THF/tol	closed	81	37	398	184
TSC	THF/tol	open	6	3	39	16

3. Stretching traces

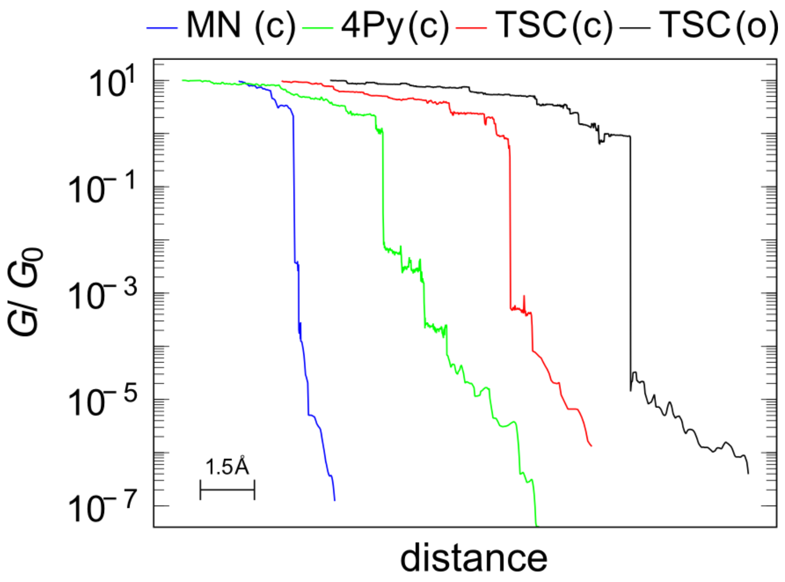

Figure S2: Examples of opening traces of photochromic molecular junctions are shown for TSC in open and closed form and for 4Py and MN in closed form.

Due to the described ageing effect of the substrates the distance scale can be estimated to a precision of 30% only. We therefore do not correlate the preferred conductance values to particular stretching distances.

4. Histograms

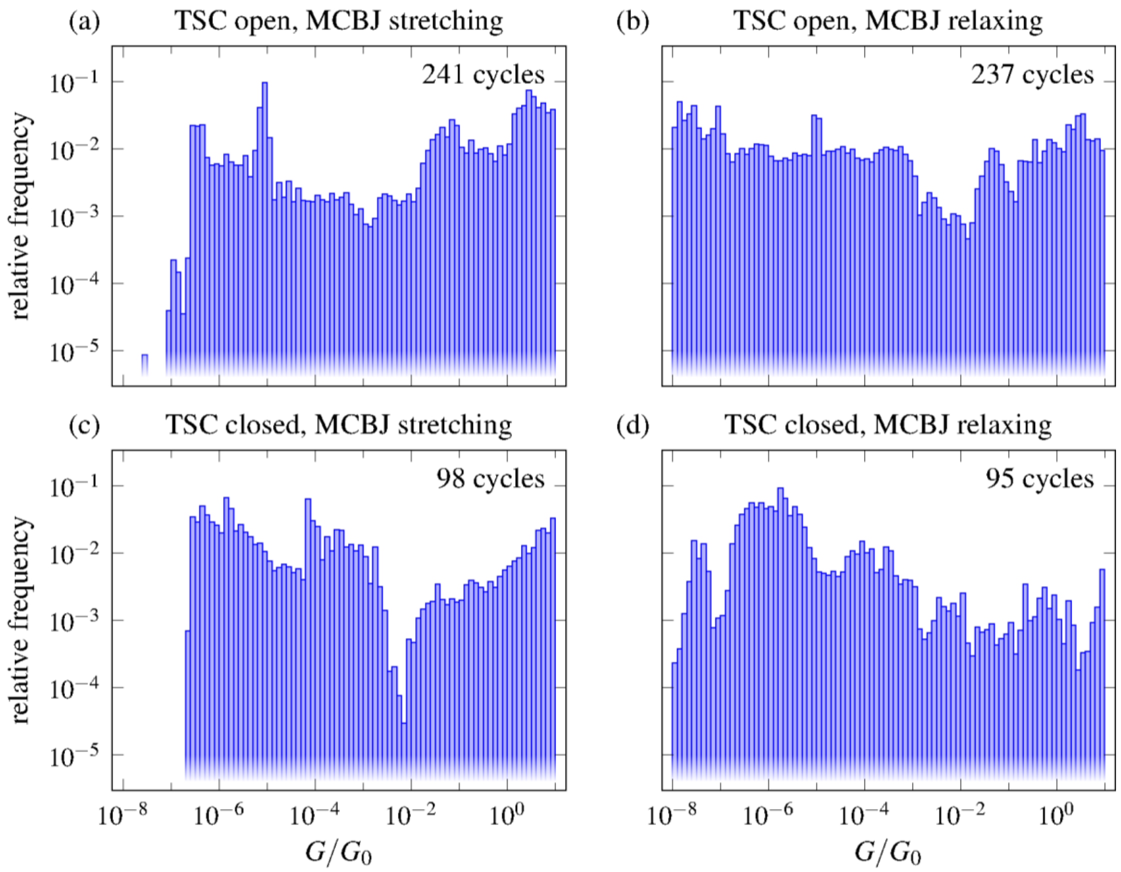

Figure S3: Histograms of stretching and relaxing curves of TSC in IPA, without interrupting the motion for recording current–voltage characteristics.

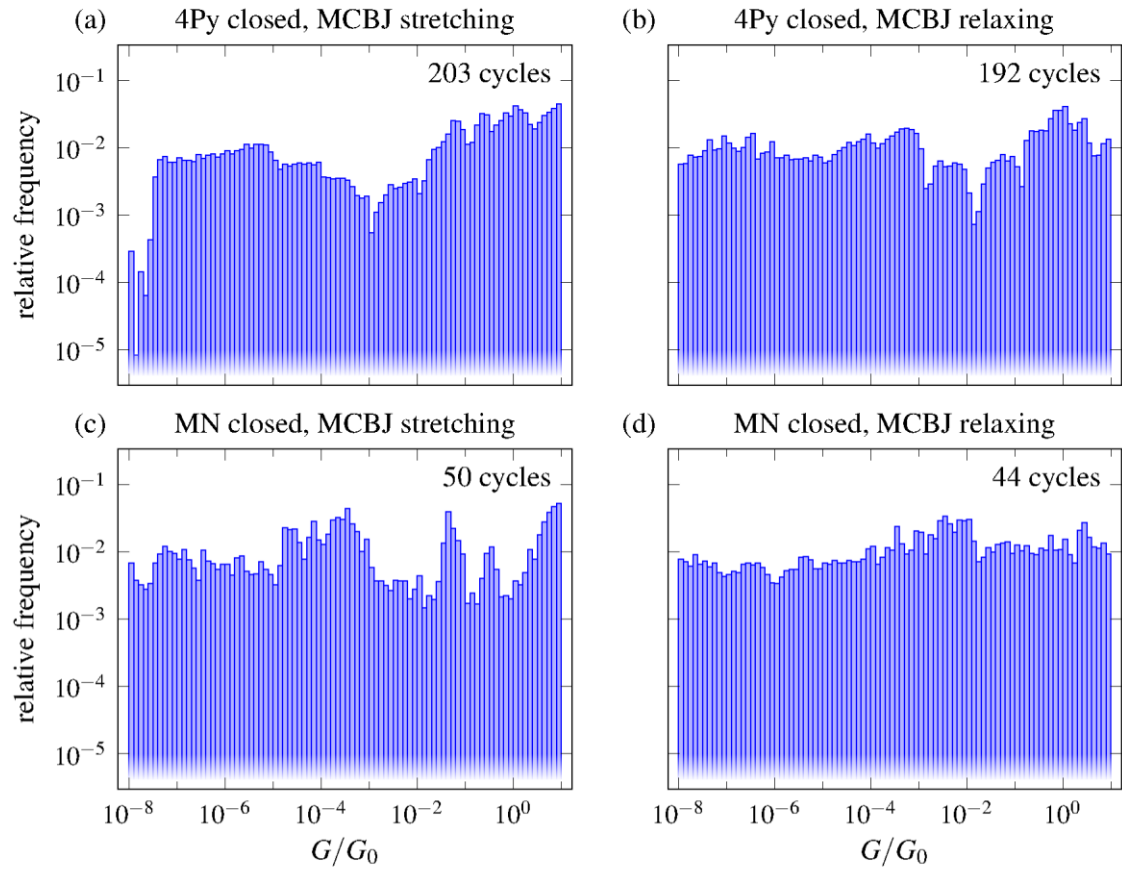

Figure S4: Histograms of stretching and relaxing curves of 4Py and MN, without interrupting the motion for recording current–voltage characteristics.

For all species we recorded continuous stretching/relaxing curves, i.e., without interruption of the motion. We applied a dc voltage of 100 mV. The initial value of the gold junctions before breaking is on the order of 300 to 500 G_0. The junctions are stretched by bending the substrate until the conductance drops below 10^{-8} G_0. The motion direction of the breaking mechanism is then reversed and the junctions are relaxed until the junction conductance arrives at a conductance of at least 100 G_0. This procedure is repeated up to a few hundred times. The MCBJ setup is optimized for maintaining single-molecule contacts constant over several minutes. Figure S3 shows the results for TSC in IPA, Figure S4 for 4Py and Mn. Junctions with conductance values covering the whole range from 10^{-7} G_0 to 10 G_0 are observed. We do not observe pronounced differences between the histograms for stretching and relaxing,

except for the fact that more data is recorded for lower conductance values $G < 10^{-7}$ G_0 when relaxing the junction. This is, however, an artefact of our measurement protocol as described above. There are no clear maxima in the histograms in the open state but an enhancement of probability for conductance values above 10^{-2} G_0. In the stretching histogram of the closed form we observe a pronounced minimum around 10^{-2} G_0. The relaxing histogram of the closed form features a higher probability for rather low conductance ~10^{-5} G_0.

5. Testing the fit procedure

To determine Γ_L, Γ_R and E_0, we fitted the measured *I*–*V* curves with the expression for the current obtained from the Landauer formula as described in Equation 1 to Equation 3 of the manuscript. The integral was numerically evaluated with the program “Octave” applying a Levenberg–Marquardt algorithm. We evaluated the Fermi functions for k_BT = 25 meV, corresponding to a temperature of 290 K.

Furthermore, we tested the quality of the fit regarding its functional dependence. As discussed in [S3], for small voltages, Equation 1 can be expanded into a sum of a linear and a cubic term of the voltage as:

$$I(V) = A_1V + A_2V^3 \quad \text{(S1)}$$

Both prefactors A_1 and A_3 depend on Γ_L, Γ_R and on E_0 in a unique way, implying a correlation between them [S3]. If the *I*–*V* does not correspond to the functional shape described by the model (Equation 1 to Equation 3), the resulting best-fit results do depend on the voltage range [S4,S5]. A helpful method to test the validity of the model is thus given by varying the fit range. We performed this procedure for our data by varying the fit range from [−0.5 V, +0.5 V] to [−1 V, 1 V]. We discarded all *I*–*V*s in which

the best-fit parameters varied by more than 20%. Finally we compared the transmission $T(E,0)$ calculated from the best-fit results with the one deduced from the linear conductance measured in a range of [−100 mV, 100 mV]. We labeled such *I–V*'s as "fittable" for which $T(E,0)_{calc}$ and $T_{lin} = G/G_0$ agree within 50% error. For MN the *I–V*'s were measured in the voltage range of [−0.5 V, +0.5 V] only. The "fittable" *I–V*'s were selected on the basis of the latter criterion only.

6. Current–voltage characteristics of the solvents Tol and THF/Tol

We performed control experiments in the pure solvents Tol and THF/Tol and occasionally also observed s-shaped *I–V*'s. As explained in section 2 these occurred after repeated stretching and relaxing of the electrodes in the junction. Stable *I–V*'s without jumps were mainly found in the high-conductance range $G > 0.01\ G_0$. While the *I–V*'s obtained shortly after starting the measurement are mainly linear in the voltage range of +/− 1 V, nonlinear *I–V*'s that were describable with the single-level model occurred after several hours of repeated stretching/relaxing and voltage sweeping. Examples are given in Figure S5. The fitting parameters of symmetric *I–V*'s are shown in Figure S6 together with those for MN and 4Py. While the coupling constants are in a similar range as for the molecular junctions, the corresponding E_0 are not, enabling us to distinguish molecular contacts from "solvent" contacts. We believe that these contacts correspond to disordered metal contacts in which residues from the solvent are incorporated into the metal, as described in [S2]. In the solvent ethanol no fittable *I–V*'s could be recorded, presumably due to the hygroscopic and polar properties of the solvent, enhancing the probability of electrochemical reactions during sweeping. Reducing the voltage range to 0.5 V or less resulted in unreliable fitting, i.e., the fit results were strongly dependent on the fit range.

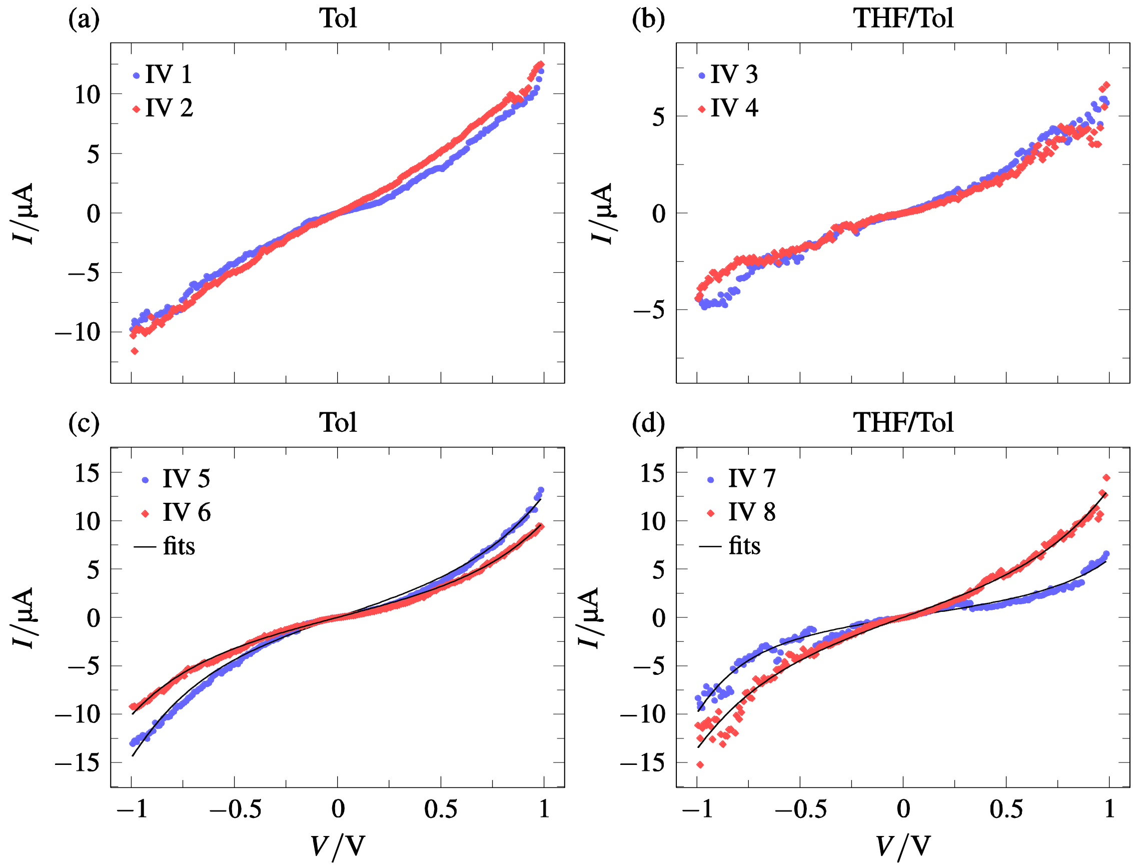

Figure S5: Examples of current–voltage characteristics (*I*–*V*'s) recorded on gold electrodes immersed with the pure solvents Tol and THF/Tol. For the examples in the upper two panels the fit with the single-level model did not converge or resulted in fitting parameters that are clearly out of the physically expected range for solvents, i.e., $\Gamma > 0.5$ eV and/or $E_0 > 1.5$ eV. They furthermore did not fulfil the criteria described in section S6, i.e., the fit parameters showed a pronounced dependence on the fit range and linear conductance was not well described by the fitted function. The fit parameters for the examples in the lower panel are given in Table S3 below.

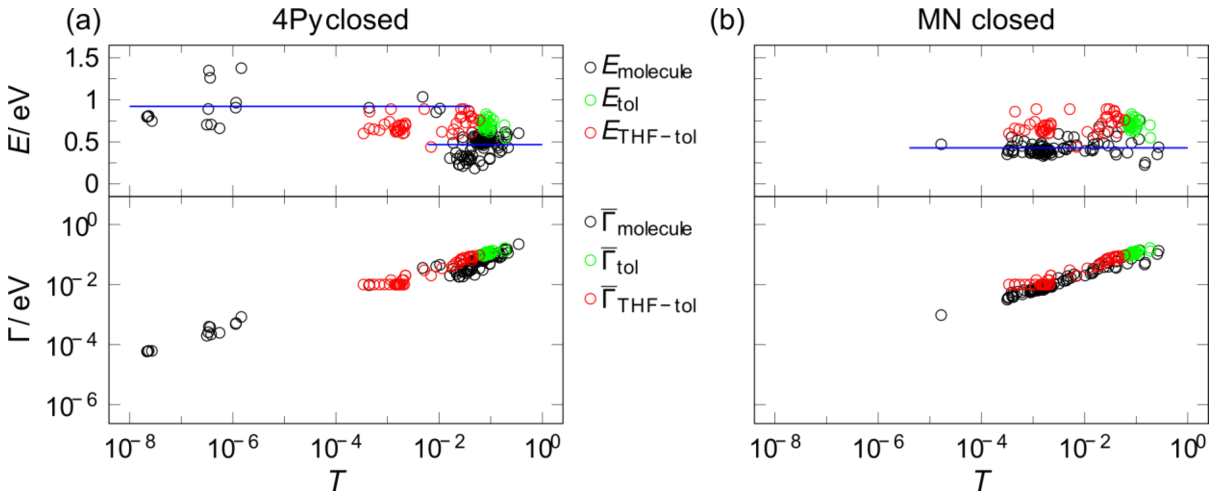

Figure S6: Fitting parameters for (a) closed form of 4Py, and (b) closed form of MN as a function of the transmission for symmetric *I*–*V*'s compared to those obtained on *I*–*V*'s that were obtained in the solvents Tol and THF/Tol.

7. Current–voltage characteristics of TSC in THF/Tol

In order to test whether the properties of the junctions that were formed depend on the solvent, we investigated the species TSC in a mixture of 50% tetrahydrofuran and 50% toluene. Within our statistics we did not find characteristic differences in the data recorded for TSC in ethanol. Figure S7 displays examples of *I*–*V*'s and their fittings with the single-level model. The fitting parameters are indicated in the figure caption and in Table S3.

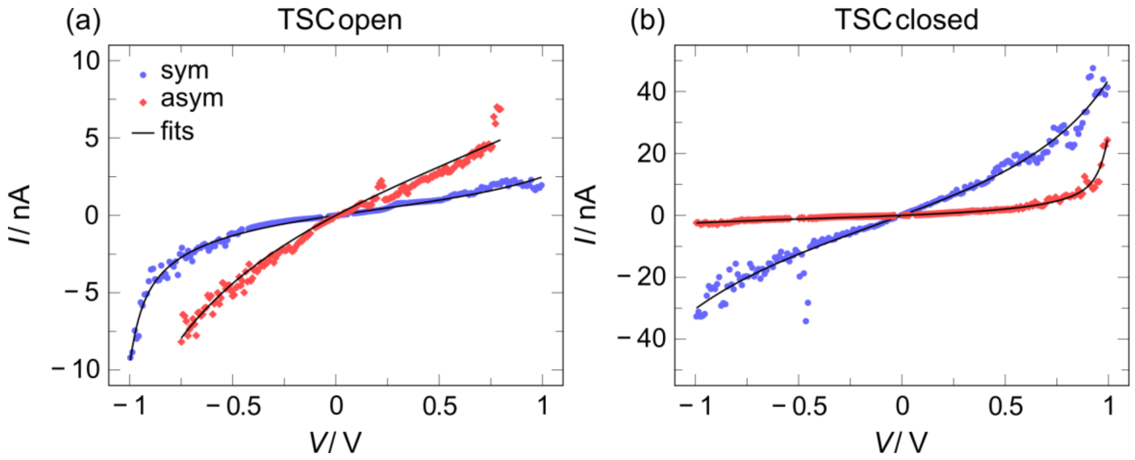

Figure S7: Examples of *I*–*V* characteristics of TSC dissolved in THF/Tol. (a) Open form, (b) closed form. The colored curves are experimental data, the black curves are the fit to the single-level model Equation 2 of the main text. The fitting parameters are: open/sym: E_0 = 0.74 eV, Γ_1 = 2.26 meV, Γ_2 = 1.55 meV; open/asym: E_0 = 1.13 eV, Γ_1 = 7.81 meV, Γ_2 = 3.67 meV; closed/sym: E_0 = 0.90 eV, Γ_1 = 7.82 meV, Γ_2 = 9.00 meV; closed/asym: E_0 = 0.74 eV, Γ_1 = 2.26 meV, Γ_2 = 1.55 meV.

8. Best-fit parameters of all current–voltage characteristics displayed in the manuscript and the Supporting Information

Table S3: Best-fit parameters of the *I*–*V* characteristics shown in Figure 2 of the manuscript and Figure S5 and Figure S7.

molecule	solvent	state/ *I*–*V*-label	symmetry	Γ_1 [eV]	Γ_2 [eV]	E_0 [eV]
4Py	Tol	closed	asym	$6.479270\cdot10^{-3}$	$5.658614\cdot10^{-1}$	$5.649362\cdot10^{-1}$
4Py	Tol	closed	sym	$5.122730\cdot10^{-2}$	$4.443719\cdot10^{-2}$	$5.555399\cdot10^{-1}$
MN	Tol	closed	asym	$2.524566\cdot10^{-2}$	$9.500361\cdot10^{-2}$	$5.379697\cdot10^{-1}$
MN	Tol	closed	sym	$6.837520\cdot10^{-3}$	$6.413143\cdot10^{-3}$	$3.327390\cdot10^{-1}$
TSC	IPA	closed	asym	$1.271382\cdot10^{-3}$	$3.557295\cdot10^{-3}$	$9.635170\cdot10^{-1}$
TSC	IPA	closed	sym	$1.892529\cdot10^{-3}$	$1.572624\cdot10^{-3}$	$6.913722\cdot10^{-1}$

TSC	IPA	open	asym	$9.895522{\cdot}10^{-4}$	$2.903467{\cdot}10^{-1}$	$1.146079{\cdot}10^{0}$
TSC	IPA	open	sym	$6.870962{\cdot}10^{-4}$	$7.353113{\cdot}10^{-3}$	$6.720923{\cdot}10^{-1}$
TSC	THF/Tol	closed	asym	$1.614467{\cdot}10^{-3}$	$3.567853{\cdot}10^{-3}$	$8.055559{\cdot}10^{-1}$
TSC	THF/Tol	closed	sym	$7.185325{\cdot}10^{-3}$	$9.004687{\cdot}10^{-3}$	$9.043714{\cdot}10^{-1}$
TSC	THF/Tol	open	asym	$7.818538{\cdot}10^{-3}$	$3.672783{\cdot}10^{-3}$	$1.129267{\cdot}10^{0}$
TSC	THF/Tol	open	sym	$2.264141{\cdot}10^{-3}$	$1.547008{\cdot}10^{-3}$	$7.418602{\cdot}10^{-1}$
none	Tol	IV5	sym	$9.64418{\cdot}10^{-2}$	$1.032215{\cdot}10^{-1}$	$6.171954{\cdot}10^{-1}$
none	Tol	IV6	sym	$9.27119{\cdot}10^{-2}$	$9.36265{\cdot}10^{-2}$	$6.681786{\cdot}10^{-1}$
none	THF/Tol	IV7	sym	$7.09558{\cdot}10^{-2}$	$5.93901{\cdot}10^{-2}$	$6.143437{\cdot}10^{-1}$
none	THF/Tol	IV8	sym	$1.06452{\cdot}10^{-1}$	$1.077149{\cdot}10^{-1}$	$6.423904{\cdot}10^{-1}$

9. X-ray single-crystal structure determination

Materials and Methods

Data collection for X-ray structure determination was performed at a *STOE IPDS-II* diffractometer equipped with a graphite monochromated radiation source (λ = 0.71073 Å), an image plate detection system, and an *Oxford Cryostream 700* with nitrogen as coolant gas. The selection, integration, and averaging procedure of the measured reflex intensities, the determination of the unit cell by a least-squares fit of the 2Θ values, data reduction, LP correction, and the space group determination were performed by using the *X-Area* software package delivered with the diffractometer. A semiempirical absorption correction method was performed after indexing of the crystal faces. The structures were solved by direct methods (*SHELXS-97*) [S3] and refined by standard Fourier techniques against F square with a full-matrix least-squares algorithm by using *SHELXL-97* and the *WinGX* (1.80.05) [S4] software package. All nonhydrogen

atoms were refined anisotropically. Hydrogen atoms were placed in calculated positions and refined with a riding model. Graphical representations were prepared with *ORTEP-III*. [S5] Crystallographic data (excluding structure factors) have been deposited with the Cambridge Crystallographic Data Centre as supplementary publication CCDC 894167 (MN). Copies of the data can be obtained free of charge on application to CCDC, 12 Union Road, Cambridge CB21EZ, U.K. (fax: (+44)1223−336−033. e-mail: deposit@ccdc.cam.ac.uk or http://www.ccdc.cam.ac.uk).

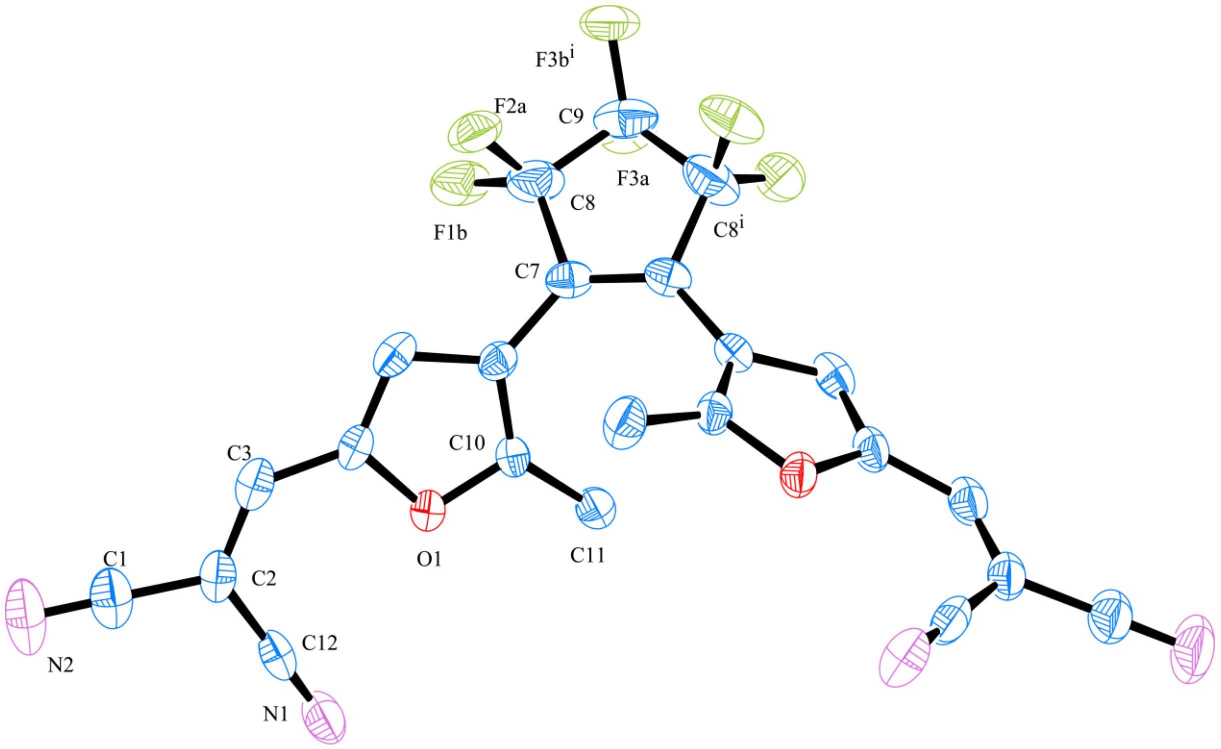

Figure S8: ORTEP-plot of MN. Disordered fluorine atoms and hydrogen atoms are omitted for clarity. Ellipsoids are plotted at the 50% probability level.

Table S4: Crystal data and structure refinement for MN.

Empirical formula	C23 H10 F6 N4 O2
Formula weight	488.35
Temperature	100(2) K
Wavelength	0.71073 Å
Crystal system	Monoclinic
Space group	C 2/c
Unit cell dimensions	a = 21.890(4) Å α= 90°.
	b = 9.2771(15) Å β= 99.344(12)°.
	c = 10.6726(14) Å γ = 90°.
Volume	2138.6(6) Å^3
Z	4
Density (calculated)	1.517 Mg/m^3
Absorption coefficient	0.135 mm^{-1}
F(000)	984
Crystal size	0.400 × 0.267 × 0.100 mm^3
Theta range for data collection	1.89 to 26.82°.
Index ranges	−27 <= h <= 27, −11 <= k <= 11, −13 <= l <= 12
Reflections collected	14031
Independent reflections	2275 [R(int) = 0.2274]
Completeness to theta = 26.82°	99.2%
Absorption correction	Integration
Max. and min. transmission	0.9871 and 0.9315
Refinement method	Full-matrix least-squares on F^2
Data / restraints / parameters	2275 / 0 / 188
Goodness-of-fit on F^2	0.985
Final R indices [I>2sigma(I)]	R1 = 0.0589, wR2 = 0.1048
R indices (all data)	R1 = 0.1270, wR2 = 0.1219
Largest diff. peak and hole	0.209 and -0.202 e.Å^{-3}

Table S5: Atomic coordinates (× 10^4) and equivalent isotropic displacement parameters (Å^2 × 10^3) for MN. U(eq) is defined as one third of the trace of the orthogonalised U^{ij} tensor.

	x	y	z	U(eq)
F(1A)	952(2)	10897(4)	3393(6)	55(2)
F(2A)	468(3)	10723(5)	701(5)	49(1)
F(3A)	325(2)	12008(7)	3674(4)	58(2)
F(1B)	1112(2)	10820(4)	2484(7)	58(2)
F(2B)	731(3)	10907(5)	1341(7)	53(1)
F(3B)	72(3)	12724(5)	3219(5)	62(2)
O(1)	1257(1)	5829(2)	2657(2)	33(1)
N(1)	2070(1)	2981(3)	3212(2)	49(1)
N(2)	3089(1)	3788(3)	94(3)	69(1)
C(1)	2707(1)	4157(3)	637(3)	48(1)
C(2)	2231(1)	4609(3)	1335(3)	36(1)
C(3)	1914(1)	5836(3)	1028(3)	37(1)
C(4)	1445(1)	6494(3)	1625(2)	32(1)
C(5)	1130(1)	7736(3)	1364(2)	35(1)
C(6)	711(1)	7866(2)	2252(2)	29(1)
C(7)	291(1)	9064(2)	2368(2)	32(1)
C(8)	505(1)	10574(3)	2197(4)	58(1)
C(9)	0	11550(4)	2500	51(1)
C(10)	812(1)	6686(3)	3030(2)	30(1)
C(11)	572(1)	6215(3)	4176(3)	41(1)
C(12)	2139(1)	3708(3)	2376(3)	37(1)

Table S6: Bond lengths [Å] for MN.

F(1A)-C(8)	1.507(5)	O(1)-C(4)	1.383(3)	C(6)-C(10)	1.370(3)
F(2A)-C(8)	1.592(7)	N(1)-C(12)	1.148(3)	C(6)-C(7)	1.461(3)
F(3A)-C(9)	1.403(4)	N(2)-C(1)	1.144(4)	C(7)-C(7)#1	1.348(5)
F(1B)-C(8)	1.334(4)	C(1)-C(2)	1.438(4)	C(7)-C(8)	1.498(4)
F(1B)-F(2B)	1.363(12)	C(2)-C(3)	1.346(4)	C(8)-C(9)	1.504(4)
F(2B)-C(8)	1.150(6)	C(2)-C(12)	1.430(4)	C(9)-F(3B)#1	1.327(5)
F(3B)-C(9)	1.327(5)	C(3)-C(4)	1.430(4)	C(9)-F(3A)#1	1.403(4)
F(3B)-F(3B)#1	1.516(11)	C(4)-C(5)	1.348(3)	C(9)-C(8)#1	1.504(4)
O(1)-C(10)	1.366(3)	C(5)-C(6)	1.427(3)	C(10)-C(11)	1.474(4)

Symmetry transformations used to generate equivalent atoms: #1 -x,y,-z+1/2.

Table S7: Angles [°] for MN.

C(8)-F(1B)-F(2B)	50.4(3)	F(2B)-C(8)-F(1A)	108.3(6)
C(8)-F(2B)-F(1B)	63.5(5)	F(2B)-C(8)-F(1A)	108.3(6)
C(9)-F(3B)-F(3B)#1	55.2(3)	C(7)-C(8)-F(1A)	104.8(3)
C(10)-O(1)-C(4)	106.92(18)	C(9)-C(8)-F(1A)	95.6(3)
N(2)-C(1)-C(2)	179.2(4)	F(1B)-C(8)-F(2A)	95.8(5)
C(3)-C(2)-C(12)	123.5(2)	C(7)-C(8)-F(2A)	103.7(3)
C(3)-C(2)-C(1)	120.6(3)	C(9)-C(8)-F(2A)	104.2(3)
C(12)-C(2)-C(1)	115.9(2)	F(1A)-C(8)-F(2A)	138.8(5)
C(2)-C(3)-C(4)	129.2(2)	F(3B)-C(9)-F(3B)#1	69.6(6)
C(5)-C(4)-O(1)	109.7(2)	F(3B)#1-C(9)-F(3A)	105.2(6)
C(5)-C(4)-C(3)	130.7(2)	F(3B)-C(9)-F(3A)#1	105.2(6)
O(1)-C(4)-C(3)	119.5(2)	F(3A)-C(9)-F(3A)#1	144.7(6)
C(4)-C(5)-C(6)	107.3(2)	F(3B)-C(9)-C(8)#1	113.1(2)
C(10)-C(6)-C(5)	106.2(2)	F(3B)#1-C(9)-C(8)#1	126.6(3)
C(10)-C(6)-C(7)	126.7(2)	F(3A)-C(9)-C(8)#1	105.9(2)
C(5)-C(6)-C(7)	127.0(2)	F(3A)#1-C(9)-C(8)#1	95.2(3)
C(7)#1-C(7)-C(6)	130.33(13)	F(3B)-C(9)-C(8)	126.6(3)
C(7)#1-C(7)-C(8)	110.52(16)	F(3B)#1-C(9)-C(8)	113.0(2)
C(6)-C(7)-C(8)	119.1(2)	F(3A)-C(9)-C(8)	95.2(3)
F(2B)-C(8)-F(1B)	66.1(6)	F(3A)#1-C(9)-C(8)	105.9(2)
F(2B)-C(8)-C(7)	122.1(4)	C(8)#1-C(9)-C(8)	106.0(3)
F(1B)-C(8)-C(7)	116.9(3)	O(1)-C(10)-C(6)	109.9(2)
F(2B)-C(8)-C(9)	115.8(3)	O(1)-C(10)-C(11)	115.8(2)
F(1B)-C(8)-C(9)	126.0(3)	C(6)-C(10)-C(11)	134.2(2)
C(7)-C(8)-C(9)	106.3(3)	N(1)-C(12)-C(2)	179.5(3)

Symmetry transformations used to generate equivalent atoms: #1 -x,y,-z+1/2.

Table S8: Anisotropic displacement parameters ($Å^2 \times 10^3$) for MN. The anisotropic displacement factor exponent takes the form: $-2\pi^2[h^2a^{*2}U^{11} + ... + 2 h k a^* b^* U^{12}]$.

	U^{11}	U^{22}	U^{33}	U^{23}	U^{13}	U^{12}
F(1A)	52(2)	45(2)	59(4)	2(2)	−15(2)	−17(2)
F(2A)	72(3)	36(2)	42(3)	10(2)	21(2)	0(2)
F(3A)	75(3)	49(3)	46(2)	−13(2)	3(2)	−18(2)
F(1B)	46(2)	37(2)	84(5)	7(2)	−4(2)	−14(2)
F(2B)	68(3)	37(2)	60(4)	10(2)	28(3)	−4(2)
F(3B)	89(3)	32(2)	67(3)	−18(2)	27(3)	−17(2)
O(1)	30(1)	34(1)	37(1)	3(1)	14(1)	2(1)
N(1)	47(1)	54(2)	48(2)	−2(1)	16(1)	17(1)
N(2)	48(2)	95(2)	72(2)	−26(2)	32(2)	−3(1)
C(1)	37(2)	60(2)	48(2)	−18(2)	13(1)	−7(1)
C(2)	29(1)	46(2)	34(2)	−9(1)	11(1)	−7(1)
C(3)	33(1)	47(2)	31(1)	−5(1)	11(1)	−11(1)
C(4)	30(1)	38(1)	31(1)	2(1)	12(1)	−7(1)
C(5)	33(1)	41(1)	32(1)	6(1)	6(1)	−11(1)
C(6)	26(1)	28(1)	32(1)	0(1)	4(1)	−6(1)
C(7)	35(1)	27(1)	31(1)	3(1)	−2(1)	−2(1)
C(8)	41(2)	36(2)	93(3)	14(2)	−1(2)	−7(1)
C(9)	84(3)	28(2)	37(2)	0	3(2)	0
C(10)	23(1)	32(1)	35(1)	1(1)	7(1)	2(1)
C(11)	43(2)	40(2)	44(2)	10(1)	18(1)	11(1)
C(12)	30(1)	44(2)	39(2)	−12(1)	9(1)	9(1)

10. References

[S1] Grüter, L.; González, M. T.; Huber, R.; Calame, M.; Schönenberger, C. *Small* **2005,** *1,* 1067–1070. doi:10.1002/smll.200500145

[S2] Hansen, K.; Nielsen, S. K.; Brandbyge, M.; Lægsgaard, E.; Stensgaard, I.; Besenbacher, F. *Appl. Phys. Lett.* **2000,** *77,* 708. doi:10.1063/1.127093

[S3] Hong, W. J.; Valkenier, H.; Mészáros, G.; Manrique, D. Z.; Mishchenko, A.; Putz, A.; García, P. M.; Lambert, C. J.; Hummelen, J. C.; Wandlowski, T. *Beilstein J. Nanotechnol.* **2011,** *2,* 699–713. doi:10.3762/bjnano.2.76

[S4] Kim, Y.; Hellmuth, T. J.; Sysoiev, D.; Pauly, F.; Pietsch, T.; Wolf, J.; Erbe, A.; Huhn, T.; Groth, U.; Steiner, U. E.; Scheer, E. *Nano Lett.* **2012,** *12,* 3736–3742. doi:10.1021/nl3015523

[S5] Wang, W. Y.; Lee, T.; Reed, M. A. *Phys. Rev. B* **2003,** *68,* 035416. doi:10.1103/PhysRevB.68.035416

[S6] G. M. Sheldrick, SHELX-97, an integrated system for solving and refining crystal structures from diffraction data; University of Göttingen: Göttingen, Germany, 1997.

[S7] Farrugia, L. J. *J. Appl. Crystallogr.* **1999,** *32,* 837–838. doi:10.1107/S0021889899006020

[S8] Farrugia, L. J. *J. Appl. Crystallogr.* **1997,** *30,* 565. doi:10.1107/S0021889897003117
